# Exogenous gibberellin altered morphology, anatomic and transcriptional regulatory networks of hormones in carrot root and shoot

**DOI:** 10.1186/s12870-015-0679-y

**Published:** 2015-12-15

**Authors:** Guang-Long Wang, Feng Que, Zhi-Sheng Xu, Feng Wang, Ai-Sheng Xiong

**Affiliations:** State Key Laboratory of Crop Genetics and Germplasm Enhancement, College of Horticulture, Nanjing Agricultural University, Nanjing, 210095 China

**Keywords:** Gibberellins, Morphology, Anatomic, Transcript profiles, Hormonal crosstalk, *Daucus carota* L

## Abstract

**Background:**

Gibberellins stimulate cell elongation and expansion during plant growth and development. Carrot is a root plant with great value and undergoes obvious alteration in organ size over the period of plant growth. However, the roles of gibberellins in carrot remain unclear.

**Results:**

To investigate the effects of gibberelliins on the growth of carrot, we treated carrot plants with gibberellic acid 3 (GA_3_) or paclobutrazol (a gibberellin inhibitor). The results found that GA_3_ dramatically reduced the root growth but stimulated the shoot growth of carrot. It also significantly promoted xylem development in the tuberous root of carrot. In addition, transcript levels of genes related to gibberellins, auxin, cytokinins, abscisic acid and brassinolides were altered in response to increased or reduced gibberellins.

**Conclusions:**

The inhibited tuberous root growth but enhanced shoot growth in plants treated with GA_3_ can be principally attributed to the changes in the xylem development of carrot roots. Negative feedback regulation mechanism of gibberellin biosynthesis also occurred in response to altered gibberellin accumulation. Gibberellins may interact with other hormones to regulate carrot plant growth through crosstalk mechanisms. This study provided novel insights into the functions of gibberellins in the growth and development of carrot.

## Background

Plant growth and development are stimulated by environmental or intrinsic cues, such as hormones [1, 2]. Gibberellins (GAs) are indispensable stimulators of plant growth [3]. These hormones are initiated from the diterpenoid pathway. To date, over 100 GAs have been identified, but only a few of them are bioactive [4]. Nowadays, the application of exogenous GAs is commonly used to regulate plant growth and development [5].

**Fig. 1:**
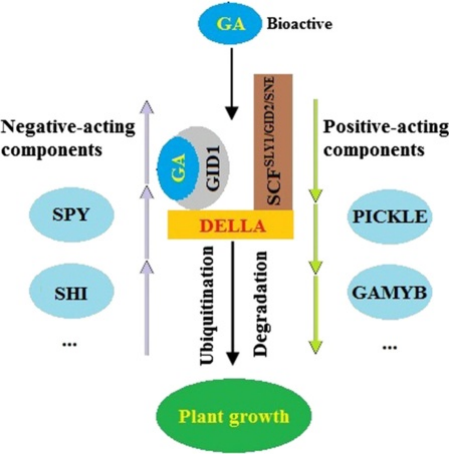
Receptors and acting components in GA response in higher plants

GA metabolism and perception have been extensively documented [6]. In vascular plants, geranyl geranyl diphosphate (GGDP) is first converted to *ent*-kaurene by *ent*-copalyl diphosphate synthase (CPS) and *ent*-kaurene synthase (KS). GA_12_ is then produced with oxidation by *ent*-kaurene oxidase (KO) and *ent*-kaurenoic acid oxidase (KAO). Stepwise oxidation is catalyzed by GA20-oxidase (GA20ox) and GA3-oxidase (GA3ox) to produce various GA intermediates and bioactive GAs, whereas GA2-oxidase (GA2ox) is involved in inactivation of bioactive GAs. GA-mediated growth is attributed to the degradation of DELLA proteins, which are the main restraints for plant growth [7]. Binding of GA to the receptor, GIBBERELLIN INSENSITIVE DWARF1 (GID1) triggers a close link between GID1 and DELLA; subsequently, DELLAs are degraded via the 26S proteasome [7, 8]. Other components, such as specific ubiquitin E3 ligase complex (SCF^SLY1/GID2/SNE^), SLEEPY1 (SLY1), PICKLE, SHORT INTERNODE (SHI) and SPINDLY (SPY), are essentially required for GA signal transduction (Fig. 1) [9]. Biochemical, molecular and genetic studies have suggested that genes involved in GA metabolic and signaling pathways are essential for GA accumulation and subsequent functions [10–12].

GAs are involved in various processes in plants. GA-deficient mutants of *Arabidopsis* and tomato cannot germinate without exogenous GAs [13]. By contrast, GA application can make environmental stimuli unnecessary and promote germination, indicating that GAs play essential roles in germination [14]. GAs can promote cell elongation, and GA_4_ is the active form that regulates shoot elongation in *Arabidopsis* [15]. GAs also participate in leaf expansion and fruit set and growth [16, 17]. Furthermore, GAs control root elongation and cell proliferation [18, 19].

Carrot (*Daucus carota* L.), a biennial plant from the Apiaceae family, is a root vegetable with enriched healthy composition [20–22]. Our previous work has focused on conducting carrot breeding and establishing genome and transcriptome database for carrot (http://apiaceae.njau.edu.cn/carrotdb/index.php) [23]. However, the role of GAs in the regulation of root plants is poorly understood because of technical reasons [24]. Hidden underground, root systems are often difficult to observe and quantify without any harm. A previous research indicated that GA is essentially required for carrot somatic embryogenesis [25]. However, another study suggested that applied GA inhibits the growth of carrot roots [26]. Thus, the functions of GAs in carrot remain unclear.

The present study aimed to investigate the effects of GA treatment on the growth and development of carrot. We attempted to gain novel insights into GA functions in carrot growth based on transcript profiles of genes involved in hormone metabolic and signaling pathways. Morphological and anatomical characteristics, along with hormone crosstalk, were also discussed to completely elucidate the roles of applied GA_3_. This study provided novel insights into GA-mediated plant growth and development in vascular plants.

## Results

### Plant growth analysis

**Fig. 2:**
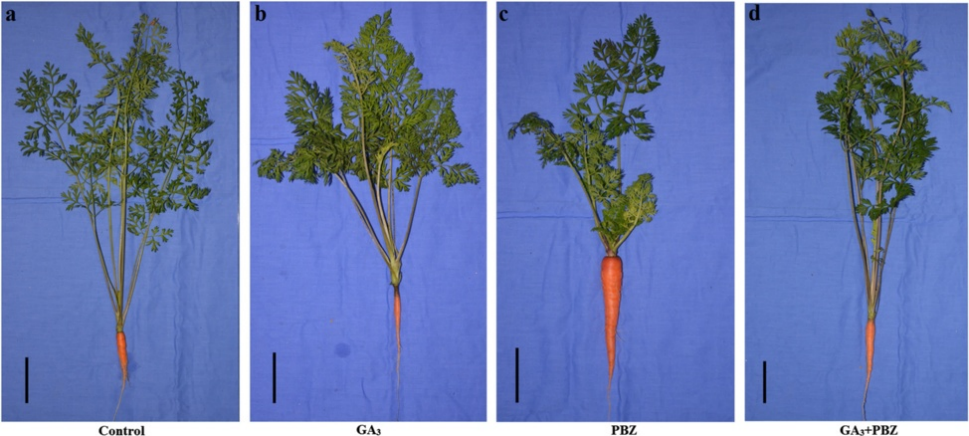
Effects of GA_3_, PBZ or GA_3_ + PBZ on carrot growth. Black lines in the lower left corner of each plant represent 5 cm in that pixel

**Fig. 3:**
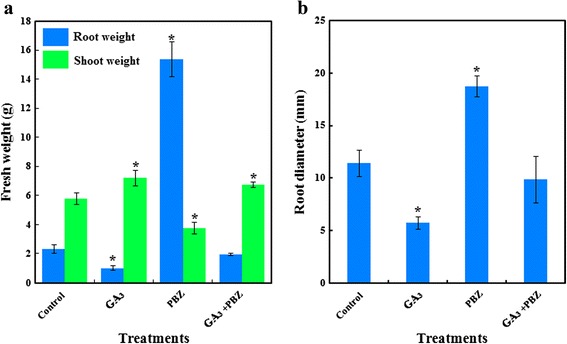
Effects of GA_3_, PBZ or GA_3_ + PBZ on carrot fresh weight (**a**) and root diameter (**b**). Values are means ± SD from three replicates, and the bars represent SD. Statistical differences were evaluated by Student *t* test at the 0.05 probability level. The asterisk indicates a significant difference compared with control group

To determine whether GA is involved in carrot plant growth, 5-week-old carrots were treated with GA or its inhibitor paclobutrazol (PBZ) weekly for five times. The effects of the applied GA and PBZ on carrot plant growth were observed after 5 weeks (Fig. 2). Exogenous GA_3_ significantly increased the shoot weight but significantly decreased root diameter and the root weight of carrot. By contrast, PBZ increased the root weight and root diameter but decreased the shoot weight of carrot. The exogenous application of GA_3_ + PBZ resulted in a phenotype similar to that of the control, which was the intermediate between the GA_3_ and the PBZ treated plants (Fig. 3).

### Anatomical structure changes in the roots, petioles and leaves

#### In the roots

**Fig. 4:**
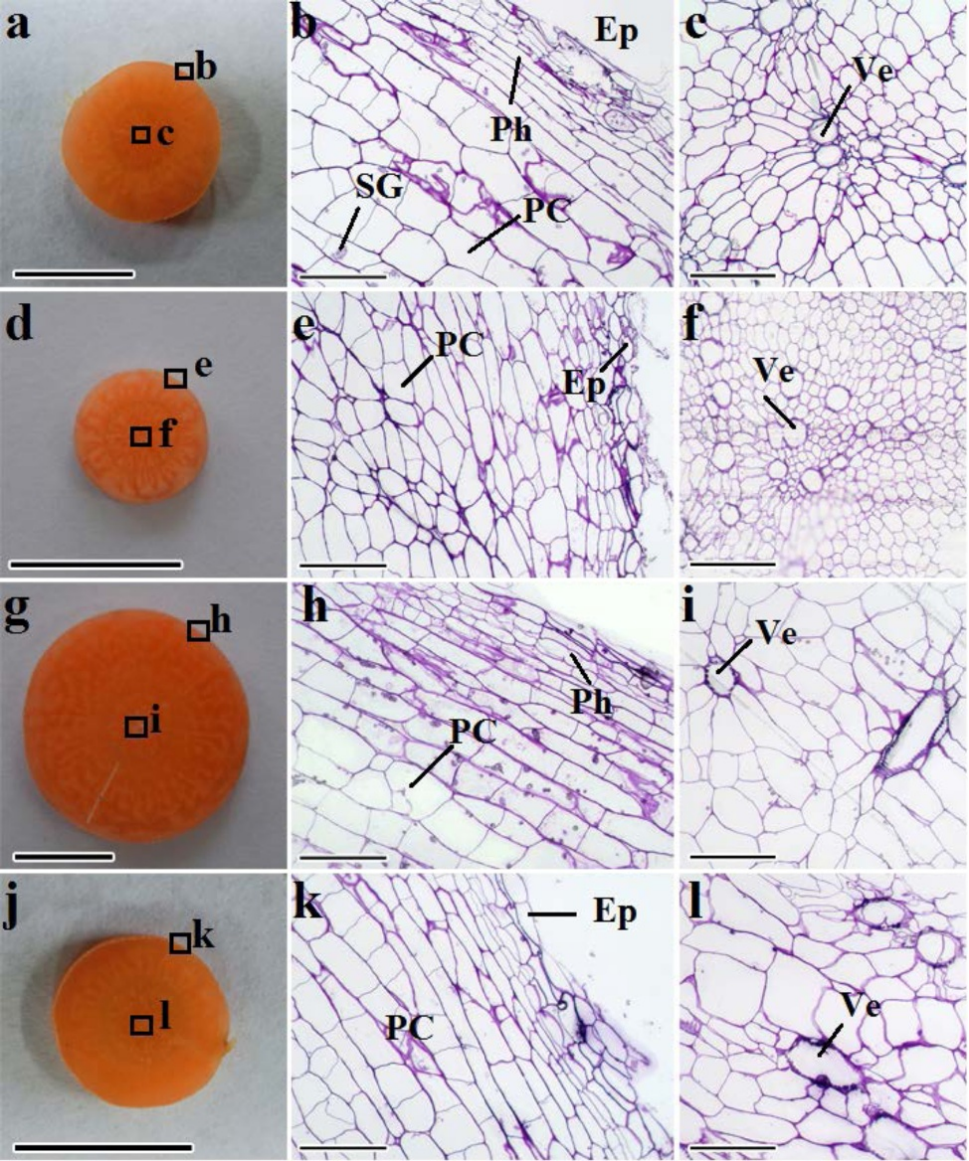
Effects of GA_3_, PBZ or GA_3_ + PBZ on the root anatomical structure of carrot. **a** (**b** and **c**), **d** (**e** and **f**), **g** (**h** and **i**) and **j** (**k** and **l**) represent the cross sections of roots under control, GA_3_, PBZ and GA_3_ + PBZ treatments, respectively. Epidermis (Ep), parenchymal cell (PC), phellogen (Ph), starch granule (SG) and vessel (Ve) are marked in the figure. Scale bars in **b**, **c**, **e**, **f**, **h**, **i**, **k** and **l** are 100 μm in length, whereas bars in **a**, **d**, **g** and **j** are 1 cm in length

**Fig. 5:**
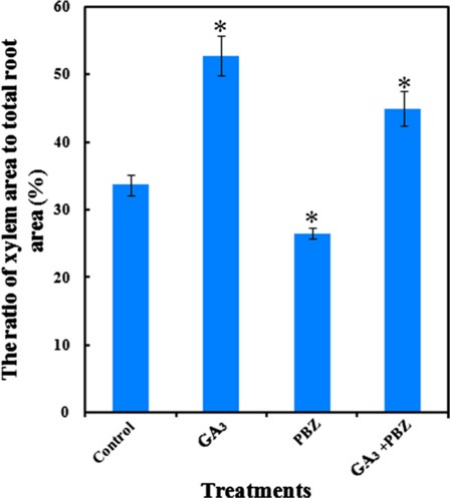
Effects of GA_3_ and PBZ treatments on the ratio of xylem area to total root area. Values are means ± SD from three replicates, and the bars represent SD. Statistical differences were evaluated by Student *t* test at the 0.05 probability level. The asterisk indicates a significant difference compared with control group

Carrot roots without any treatment were approximately 1.2 cm in diameter (Fig. 4a). GA_3_ dramatically reduced the root diameter, which was slightly relieved in the presence of PBZ (Figs. 3 and 4). Interestingly, the ratio of xylem area to total root area was significantly higher under GA_3_ treatment compared with control conditions (Fig. 5). PBZ alone significantly increased the thickness of root diameter, but decreased the ratio of xylem area to total area, which was relieved by application of GA_3_ (Figs. 3, 4 and 5).

#### In the petioles

**Fig. 6:**
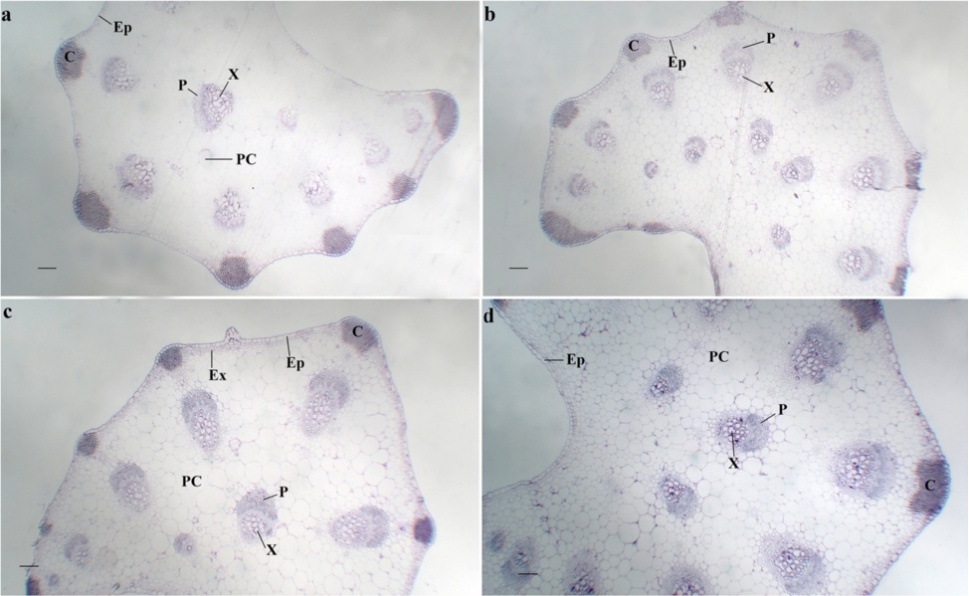
Effects of GA_3_, PBZ or GA_3_ + PBZ on the anatomical structure in carrot petioles. **a**, **b**, **c** and **d** represent the cross sections of petioles under control, GA_3_, PBZ and GA_3_ + PBZ treatments, respectively. Collenchyma (C), epidermis (Ep), exodermis (Ex), phloem (P) and xylem (X) are marked in the figure. Scale bars in **a**, **b**, **c**, **d** and e are 40 μm in length

**Fig. 7:**
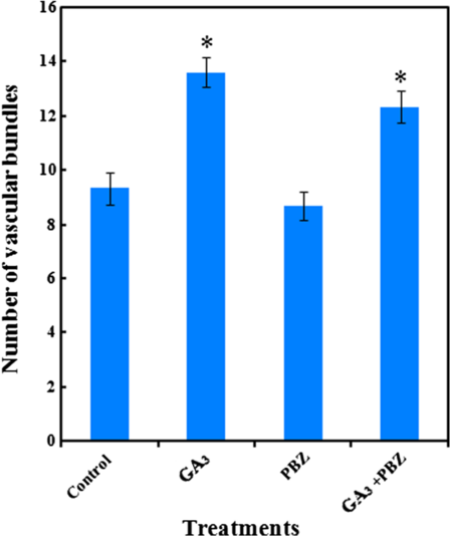
Number of vascular bundles in carrot petioles under different treatments. Data were calculated and presented as mean ± SD. Student’s *t* test was used to determine the difference between two treatments. * *P* < 0.05 was considered to be statistically significant compared with control group

GA_3_ increased the number of vascular bundles in the petioles, which may contribute to influxes of nutrients and water towards the leaves (Fig. 6b). This effect was also observed when GA_3_ was applied together with PBZ. However, PBZ did not evidently change the number of vascular bundles (Figs. 6 and 7).

#### In the leaves

**Fig. 8:**
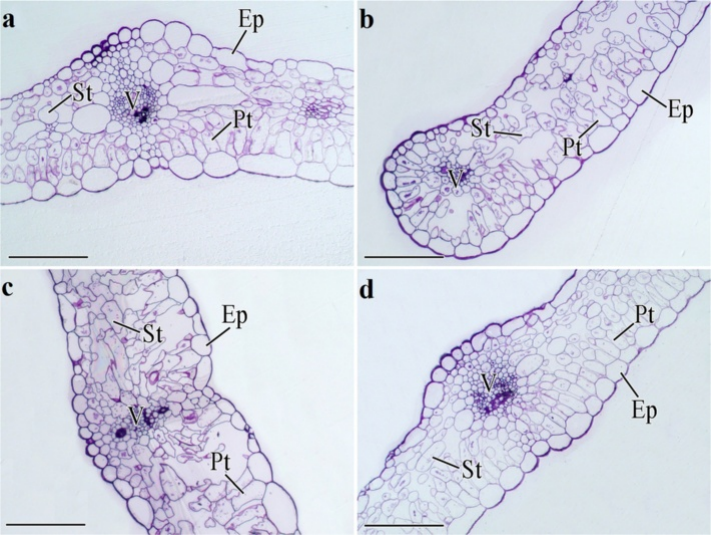
Effects of GA_3_, PBZ or GA_3_ + PBZ on the anatomical structure in carrot leaves. **a**, **b**, **c** and **d** represent the cross sections of leaves under control, GA_3_, PBZ and GA_3_ + PBZ treatments, respectively. Epidermis (Ep), palisade tissue (Pt), spongy tissue (St) and vascular (V) are marked in the figure

Palisade tissue (Pt) and spongy tissue (St) are two main components of carrot leaves. Pt, which contains the largest number of chloroplasts, may be the principal site for photosynthesis in the leaves. St is another leaf tissue that facilitates gas exchange. However, no obvious difference was detected in the anatomical structure of the leaves under different treatments (Fig. 8).

### Effects of GA_3_ treatment on the expression levels of GA biosynthetic pathway genes

**Fig. 9:**
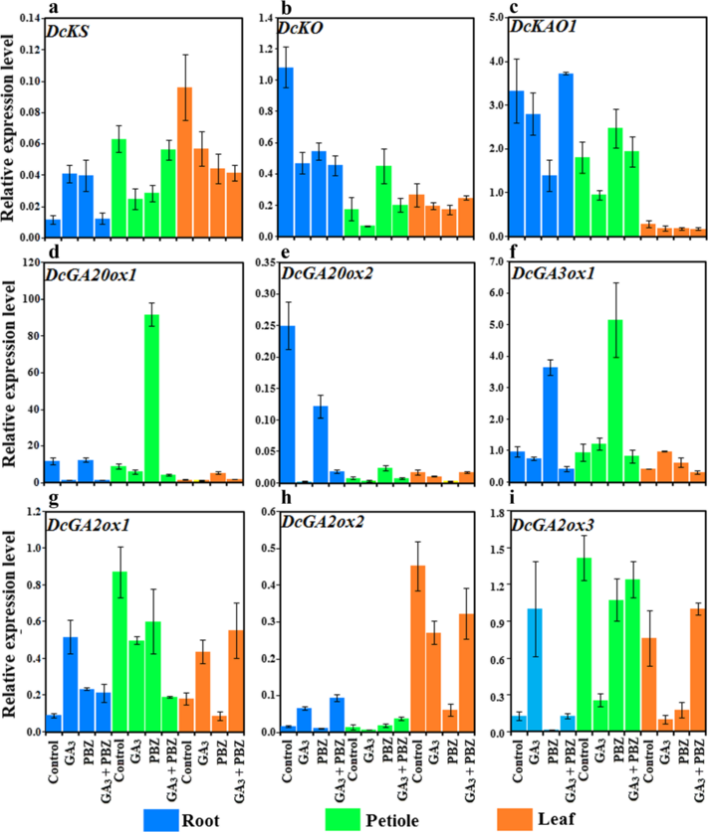
Effects of GA_3_, PBZ or GA_3_ + PBZ on the expression levels of GA pathway-related genes. Error bars represent standard errors among three independent replicates. Data are the means ± SD of three replicates

To illustrate the effects of gibberellin application on GA biosynthesis, we investigated the changes in the expression levels of GA metabolic genes. *DcKS*, *DcKO*, *DcKAO1*, *DcGA20ox1*, *DcGA20ox2*, *DcGA3ox1*, *DcGA2ox1*, *DcGA2ox2* and *DcGA2ox3* were annotated as GA pathway-related genes based on carrotDB, a transcriptomic and genomic database for carrot (Table 1). The expression levels of these selected genes were measured by quantitative real-time PCR (qRT-PCR). The biosynthetic pathway-related genes were strongly regulated by GA or PBZ treatment (Fig. 9).

**Table 1:** Nucleotide sequences of primers specific to gibberellin biosynthetic and signaling genes used for qRT-PCR

Gene	Molecular function	Gene ID in carrotDB	Primer sequences (forward/reverse)
*DcKS*	*ent*-kaurene synthase	comp49567	GCGATGGGATGTTGGCGAAGAA/CCGATTGGTGAACTCTGATTGTTGTC
*DcKO*	*ent*-kaurene oxidase	comp32899	ATGGTCGCAACAAGTGATTATGATGAG/TCTCTGTTATTACGATGTCGCTTCTGA
*DcKAO1*	*ent*-kaurenoic acid oxidase	comp50166	CACAAGCGGCTGAGACGATTAACA/TTCGACCACTTATCCAATGCAGACTT
*DcGA20ox1*	Gibberellin 20-oxidase	comp575	CCGACCTCACTCACCATTCTTCAC/CCATCTTGTTCACCACTGCTCTGT
*DcGA20ox2*	Gibberellin 20-oxidase	comp43121	AACCTAATATCGGATGCTCACAAGTCT/AGGTGGATGAGGTCTTCTTAGTAGAGT
*DcGA3ox1*	Gibberellin 3-oxidase	comp40044	GGAAGAAATGGGATGGGTCACTGT/CCGTTGGTTAGTATGTGGAGCAGAT
*DcGA2ox1*	Gibberellin 2-oxidase	comp30452	TTCAGTTCCAGCAGACCAAGACTC/GCTTGAGCAGTGAAGGCAATGG
*DcGA2ox2*	Gibberellin 2-oxidase	comp44237	TGTTGATGACTGCCTACAGGTAATGAC/CATGAGTGAAGTTGATGGTGCAATCTT
*DcGA2ox3*	Gibberellin 2-oxidase	comp47688	ACTTATAATCAGAGCCTGCGAAGAACA/GGAAGGATTGGCGTCAAGTAAGAGAT
*DcGID1b*	Gibberellin receptor GID1B	comp427507	ATGCTTCGCCGTCCTGATGG/GCTGACCTATAAACACGATTGAGAAG
*DcGID1c*	Gibberellin receptor GID1C	comp427506	AACATGCTTCGCCGTCCTGATG/GAACTGCGTTGGGAGGGACTTTG
*DcDELLA*	DELLA protein GAI	comp43703	TTGAGCGACACGAGACACTGACT/GAGGTAGCAATAAGCGAGCGAGTG
*DcSLY1*	F-box protein GID2	comp28764	GATAATTTCGCCGACAATTTCGCTGAT/GCCGTCTTGTTCCACTGCTTGT
*DcPICKLE1*	CHD3-type chromatin-remodeling factor PICKLE	comp46359	ATGTCCAACTGCTGCTGCTGATAG/TTCCACTTCACAAGATACTGCTTCACA
*DcPICKLE2*	CHD3-type chromatin-remodeling factor PICKLE	comp48322	AAGCGAGCTAGAACGAAGACAACC/CGATGGACTGAGTGAGATGAGATGAC
*DcSPY*	UDP-N-acetylglucosamine--peptide N-acetylglucosaminyltransferase SPINDLY	comp47859	TGGAGAGTTGGAGTCTGCTATCACT/AATATGCCACGCCTTGGTTAATATCG
*DcGAMYB*	Transcription factor GAMYB	comp43195	ACTATTCCAGCCAGTTGACTTCTCCT/GCGTCGTCTAATGAACTTCCACTAACA
*DcSHI*	short internodes	comp46084	GGCAACCAAGCGAAGAAGGATTGTATA/CCAAGAATGTTCACCTGCTGTCTCT

In the roots, exogenous GA_3_ application upregulated *DcKS*, *DcGA2ox1*, *DcGA2ox2* and *DcGA2ox3* expression but reduced the mRNA levels of *DcKO*, *DcGA20ox1* and *DcGA20ox2*. Similarly, GA_3_ + PBZ treatment markedly decreased the expression levels of *DcKO*, *DcGA20ox1* and *DcGA20ox2* but obviously increased that of *DcGA2ox2*. In the petioles, exogenous GA_3_ application reduced the mRNA levels of *DcKS*, *DcKO*, *DcKAO1*, *DcGA2ox1*, *DcGA2ox2* and *DcGA2ox3* but did not significantly alter the expression levels of *DcGA20ox1*, *DcGA20ox2* and *DcGA3ox1*. PBZ alone upregulated *DcKO*, *DcKAO1*, *DcGA20ox1*, *DcGA20ox2*, and *DcGA3ox1* but downregulated *DcGA2ox1* and *DcGA2ox3*. In the leaves, GA_3_ application upregulated *DcGA2ox1* but downregulated *DcKS* and *DcGA20ox2* expression. PBZ alone upregulated *DcGA20ox1* but downregulated *DcGA2ox2* and *DcGA2ox3* (Fig. 9).

### Effects of GA_3_ application on the expression levels of GA response genes

**Fig. 10:**
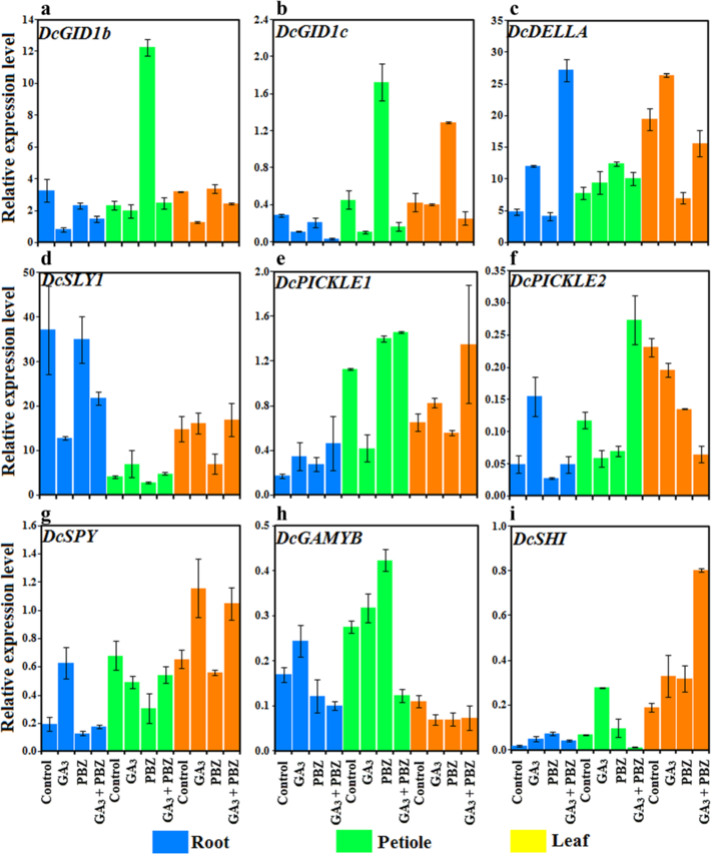
Effects of GA_3_, PBZ or GA_3_ + PBZ on the expression levels of genes involved in GA signaling. Error bars represent the standard errors among three independent replicates. Data are the means ± SD of three replicates

The proteins encoded by *DcGID1b*, *DcGID1c*, *DcDELLA*, *DcSLY1*, *DcPICKLE1*, *DcPICKLE2*, *DcSPY*, *DcGAMYB* and *DcSHI* were recognized as GA receptors or acting components by carrotDB. Thus, these genes were selected and investigated for qRT-PCR analysis (Fig. 10).

In the roots, GA_3_ treatment downregulated *DcGID1b* and *DcSLY1* but upregulated *DcDELLA*, *DcSPY* and *DcSHI*. In the petioles, GA_3_ treament alone downregulated *DcGID1c* and *DcPICKLE1*. By contrast, *DcGID1b*, *DcGID1c* and *DcPICKLE1* showed increased expression after PBZ treatment. In the leaves, exogenous GA_3_ upregulated *DELLA* and *DcSPY*. PBZ increased *DcGID1c* but suppressed *DcDELLA* (Fig. 10).

### Effects of GA_3_ application on the expression levels of genes implicated in other hormone pathway

**Fig. 11:**
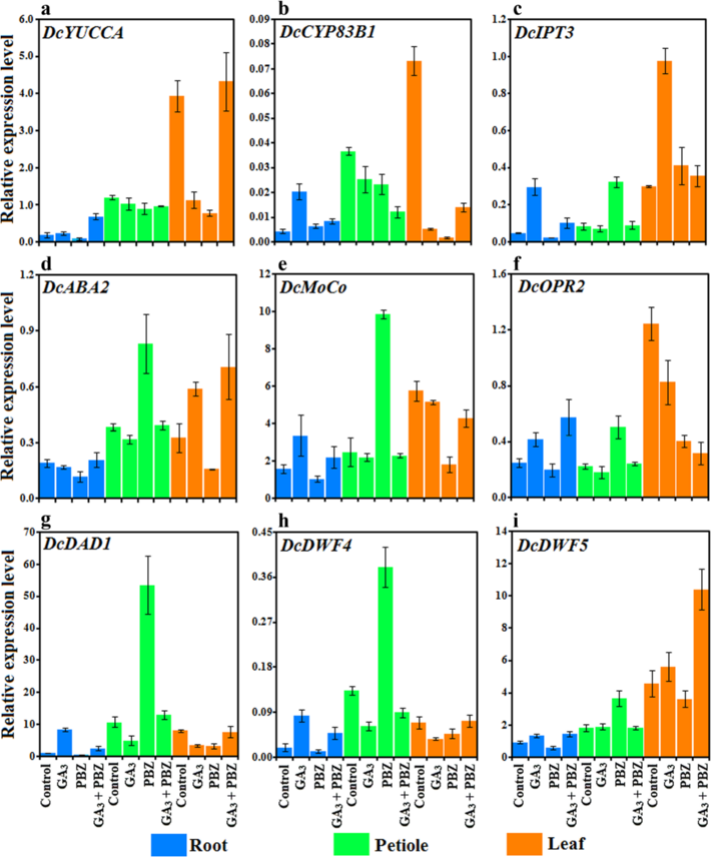
Effects of GA_3_, PBZ or GA_3_ + PBZ on the expression levels of genes involved in auxin, cytokinin, abscisic acid and brassinolide metabolism. Error bars represent the standard errors among three independent replicates. Data are the means ± SD of three replicates

To verify whether GA_3_ altered metabolism of other hormones, a total of 9 genes from auxin, cytokinin, abscisic acid, brassinolide biosynthetic pathways were identified and their expression under GA_3_ and PBZ treatments was determined (Table 2; Fig. 11). In carrot roots, transcript levels of most genes were upregulated by GA_3_ treatment. By contrast, inhibited transcription was detected when PBZ was applied, which was ameliorated by application of GA_3_. In the petioles, PBZ resulted in obvious increases in transcript levels of *DcIPT3*, *DcABA2*, *DcMoCo*, *DcOPR2*, *DcDAD1*, *DcDWF4* and *DcDWF5*, which was quite different from that in the roots. In the leaves, GA_3_ application increased transcription of *DcIPT3*, *DcABA2* and *DcDWF5* but reduced the mRNA abundance of *DcYUCCA*, *DcCYP83B1*, *DcOPR2*, *DcDAD1* and *DcDWF4* (Fig. 11).

**Table 2:** Description of genes implicated in auxin, cytokinin, abscisic acid, brassinolide biosynthesis and primers used for qRT-PCR

Gene	Molecular function	Gene ID in carrotDB	Primer sequences (forward/reverse)
*DcYUCCA*	Indole-3-pyruvate monooxygenase	comp48938	GTTCTGTCCAGTCCGAGGTTGAG/CCTCTCCTCCGAACTCTTGTAATCC
*DcCYP83B1*	Cytochrome P450 83B1	comp43558	GACATGCTGAGTACGGCAGTTACC/TCCAATGAATGGAAGTCCAGGAGGA
*DcIPT3*	Adenylate isopentenyltransferase	comp34617	GAATGGAATGGTAGATGAGGCAAGACA/TCTCTAACTGGCGGCAGGCTAG
*DcABA2*	Xanthoxin dehydrogenase	comp50471	CGATATTATGGTCAACAATGCGGGTAT/CGTCGTGCTGCTTACACTACTCA
*DcMoCo*	MoCo sulfurase	comp15442	CCTGGAACTGATTGGAATACCGAAGT/GCCTGGATTAATGGAATAGACGCCTTA
*DcOPR2*	OPDA reductase	comp45214	CACATGGTGGAGCCAAGGATGAA/AGCAACAAGGTCAGTACAGTTCTCAG
*DcDAD1*	Lipase	comp46390	CTCGAAGAGGAGCTTAGTGAAGTTGTT/TTAGGAAGAAGAAGGACTCGGCAATAC
*DcDWF4*	Cytochrome P450 90B1	comp42688	AAACGCTAAGGCTGGGCAATGT/GCACGGCTGCAATCACTGGAA
*DcDWF5*	Sterol delta-7 reductase	comp15451	AGATGGTGGTGAAGGAGGAGAA/CACGCAGTCATAGTGGGTTTTG

## Discussion

Modifying plant growth, stature and yield has been a farming goal in professional agronomy and horticulture [27]. The use of plant growth regulators, such as synthetic hormones, has achieved great progress in recent years [28, 29]. GAs, a class of plant hormones, have been widely used to regulate seed germination, plant growth and fruit yield [30–32]. The root crop carrot is popular worldwide and has great value [33]. Thus, regulating the root and shoot growth of carrot to increase its yield has been a critical goal. The results of the present study provided new insights into the roles of GA in the growth and development of carrot.

GAs stimulate cell elongation, and this effect has been well studied in GA-deficient mutants [34, 35]. Previous studies on GAs focused on aerial parts because the root is not always economically important. However, the roots of some plants, such as carrot, need intensive attention. The results of the present study showed that GA_3_ application increased shoot growth but impaired root growth in carrot (Figs. 2 and 3), which is in agreement with the results obtained by Michel-Wolwertz and his collegues [26]. In higher plants, GA could promote cell elongation at the expense of lateral expansion [36]. Here, enlargement of the GA-treated roots was suppressed, again supporting this statement. This treatment also dramatically altered matter distribution. Therefore, excessive GA_3_ negatively controls root growth in carrot. This interesting observation agrees with a previous research in carrot [26], although some studies indicate that GAs play essential roles in root growth [37, 38].

Xylem is an important tissue for water and solute transport; this tissue also provides structural support [39]. However, the phloem tissue in carrot root provides more nutrients and metabolites than the xylem tissue [40]. As a result, an appropriate phloem/xylem ratio in carrot root is important. A previous study indicated that mobile GA promotes xylem expansion in the hypocotyl of *Arabidopsis* [41]. Similarly, the present study found that the xylem region in carrot roots treated with GA_3_ or GA_3_ + PBZ was evidently multiplied (Fig. 5). This alteration may weaken the root taste, texture and even quality.

GA accumulation within plants may mostly be regulated by biosynthetic genes, and the signals are perceived by receptors and related acting components (Fig. 1) [9, 42]. In the present research, we observed a feedback regulation of GA-related genes. GA_3_ application decreased the transcript levels of genes encoding GA20-oxidase and GA3-oxidase but increased those of genes encoding GA2-oxidase, whereas PBZ induced opposite effects on these genes. Indeed, feedback regulation of GA biosynthesis is firmly established as a mechanism to maintain GA homeostasis in higher plants [10, 43].

Treatment with GA_3_ or its inhibitor PBZ can elevate or reduce GA accumulation, thus exerting different effects on plant growth and development [44, 45]. However, we cannot attribute all the alterations to the changing levels of GAs. Indeed, GA-mediated plant growth often involves complex interaction among hormones [46]. Previous studies revealed that altered GA levels can influence accumulation, signaling, transport and even functions of other hormones [47, 48]. In this study, GA_3_ induced obvious changes in expression of hormone-related genes, suggesting GAs may interact with other hormones to regulate carrot plant growth through crosstalk mechanisms [49–51]. In addition, there seems to be an organ-specific regulation of hormone-related genes in response to GA_3_ or PBZ. For example, *DcGA2ox1* was higher expressed after GA_3_ treatment in carrot roots and leaves, but was reduced in the petioles. All these results together suggested that hormonal regulation of plant growth is a complicated regulatory network.

## Conclusions

In the present study, GA_3_ or PBZ treatment altered the morphological parameters, anatomical structure and transcriptional regulatory networks of hormones in carrot plants. GA_3_ treatment restrained root growth but enhanced shoot growth possibly because of thickened xylem region in the roots and increased area of vascular bundles in the petioles. Excessive or reduced gibberellin also altered hormone homeostasis by changing transcription of related genes, thus exerting effects on carrot plant growth.

## Methods

### Plant material and GA_3_ application

The seeds of the carrot cultivar ‘Kurodagosun’ were sown in an artificial chamber at the Nanjing Agricultural University (32°02′N, 118°50′E). The artificial weather was controlled at 25 °C for 16 h during daytime with a light intensity of 300 μmol m^−2^s^−1^ followed by 18 °C for 8 h at night. Plants were grown in pots (30 × 30 cm) filled with a mixture of vermiculite and organic soil (1:1, v/v). Five weeks after emergence, soils in containers were irrigated with 200 mL of aqueous solution that contains GA_3_ (150 ppm), PBZ (20 ppm), or the combination of both. The plants treated with aqueous solution were used as the control. All the treatments were performed weekly until the fifth week. The samples were replicated three times and harvested after the treatments. Then, the samples were morphologically characterized before storing at −80 °C until analysis.

### Anatomical structure analysis

To examine the effects of GA_3_ or PBZ treatment on carrot growth, the anatomical structure of the plants was investigated. Fresh samples were cut into small pieces of approximately 1 mm^3^ and then immediately stored in phosphate buffer solution (pH 7.2) containing 2.5 % glutaraldehyde. The slices were dehydrated with gradient ethanol and then infiltrated with epoxy propane. For embedding, the samples were placed and soaked in Spurr resin [52]. A Leica ultramicrotome (Germany) was used to cut the samples into thin sections (~1 μm). The sections were then stained with 0.5 % methyl violet for 10 min. Subsequently, the slices were placed under a Leica DMLB microscope (Germany) for observation and taking photographs.

### Total RNA isolation and cDNA synthesis

Total RNA was strictly extracted from carrot roots, petioles and leaves using an RNA extraction kit (Tiangen, Beijing, China) in accordance with the manufacturer’s directions. RNA was quantified by a One-Drop™ spectrophotometer. Total RNA was treated with gDNA Eraser for 2 min at 42 °C (TaKaRa, Dalian, China) to eliminate genomic DNA contaminants. First-strand cDNA was synthesized from the isolated RNA using a PrimeScript RT reagent kit (TaKaRa, Dalian, China) in accordance with the manufacturer’s specifications. The cDNA reaction mixture was diluted 10-fold in deionized water for qRT-PCR analysis.

### Gene expression analysis by quantitative real-time PCR

Genes involved in GA, auxin, cytokinin, abcisic acid and brassinolide pathways were selected from carrotDB (http://apiaceae.njau.edu.cn/carrotdb/index.php) [23]. qRT-PCR was performed using TaKaRa SYBR Premix *Ex Taq* (Takara, Dalian, China) in a total of volume of 20 μL. All PCR reaction mixtures contained 10 μL of SYBR Premix *Ex Taq*, 7.4 μL of deionized water, 0.4 μL of each forward and reverse primer, and 2 μL of diluted cDNA strand. PCR cycling was performed using a program of 95 °C for 30 s, followed by 40 cycles of 95 °C for 5 s and 60 °C for 30 s. The experiments were performed with three independent biological replicates and the results were normalized against carrot reference gene *DcACTIN* [53]. Data from *DcGA2ox3* in the leaves of carrot grown under GA + PBZ treatment were selected as calibrator for gene expression analysis. The PCR primer pairs of all genes are shown in Tables 1 and 2.

### Statistical analysis

Student *t* test was applied to detect differences under different treatments at the 0.05 significance level.

## Availability of supporting data

The data supporting the results of this article are included within the article.

## Abbreviations

C: collenchyma
CPS: *ent*-copalyl diphosphate synthase
Ep: epidermis
Ex: exodermis
GA: gibberellin
GA20ox: GA20-oxidase
GA2ox: GA2-oxidase
GA3ox: GA3-oxidase
GGDP: geranyl geranyl diphosphate
GID1: gibberellin Insensitive Dwarf1
KAO: *ent*-kaurenoic acid oxidase
KO: *ent*-kaurene oxidase
KS: *ent*-kaurene synthase
P: phloem
PBZ: paclobutrazol
PC: parenchymal cell
Ph: phellogen
Pt: palisade tissue
qRT-PCR: quantitative real-time PCR
SG: starch granule
SHI: short internode
SLY1: sleepy1
SPY: spindly
St: spongy tissue
V: vascular
Ve: vessel
X: xylem
